# Effects of packinghouse operations on the flavor of ‘Orri’ mandarins

**DOI:** 10.1002/fsn3.2778

**Published:** 2022-03-11

**Authors:** James Otieno, Abiola Owoyemi, Livnat Goldenberg, Yossi Yaniv, Nir Carmi, Ron Porat

**Affiliations:** ^1^ Department of Postharvest Science of Fresh Produce ARO The Volcani Institute Rishon LeZion Israel; ^2^ The Robert H Smith Faculty of Agricultural, Food and Environmental Quality Sciences The Hebrew University of Jerusalem Rehovot Israel; ^3^ Department of Fruit Tree Sciences ARO The Volcani Institute Rishon LeZion Israel

**Keywords:** citrus, flavor, mandarin, packinghouse, wax

## Abstract

Mandarins have a delicate flavor and are easy to peel and easy to consume. However, they are relatively perishable and suffer from flavor deterioration after harvest. The goal of the current study was to examine the effects of commercial packinghouse operations on the flavor of ‘Orri’ mandarins. For that purpose, we collected fruit from four different points along a commercial citrus packing line: (1) directly from the harvest bin, (2) after application of a hot (53°C) fungicide treatment for 30 s, (3) after waxing, and (4) after waxing and after the fruit had passed through a hot‐air drying tunnel (37°C) for 2 min. The collected fruit were stored for 3 or 6 weeks at 5°C and then kept for five more days under shelf‐life conditions at 22°C. The observed results indicate that the hot fungicide treatment had no effect on flavor quality. However, the waxing and waxing +drying treatments resulted in significant increases in ethanol accumulation, lower flavor‐acceptability scores, and increased off‐flavors. Gas‐chromatography mass‐spectrometry (GC–MS) analysis revealed that the waxing and waxing +drying treatments resulted in particular increases in the levels of alcohol and ethyl ester volatiles; whereas levels of other aroma volatiles (i.e., esters, aldehydes, monoterpenes, and sesquiterpenes) decreased after storage in all fruit samples. Overall, the waxing process in commercial citrus packinghouses increased ethanol and ethyl ester volatile levels and harmed flavor acceptability. These findings demonstrate the need to identify new wax formulations that do not hamper fruit‐flavor quality.

## Graphical abstract

The goal of this study was to examine the effects of commercial packinghouse operations, including the application of hot fungicides, waxing, and drying, on the flavor of ‘Orri’ mandarins. The key finding is that commercial waxing significantly increased ethanol and ethyl ester volatile levels, and somewhat harmed fruit‐flavor acceptability and increased off‐flavor sensations.

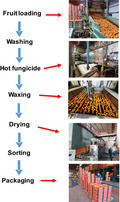

## 1 INTRODUCTION

Mandarins have a delicate flavor and are easy to consume (Goldenberg et al., 2018). However, mandarins are also much more perishable than other citrus fruit and have shorter postharvest storage lives (Cohen, 1999; Kader, 2002). Major problems in maintaining mandarin fruit quality after harvest are the decrease in flavor acceptability and the accumulation of off‐flavors over time (Tietel et al., 2011).

**FIGURE 1:**
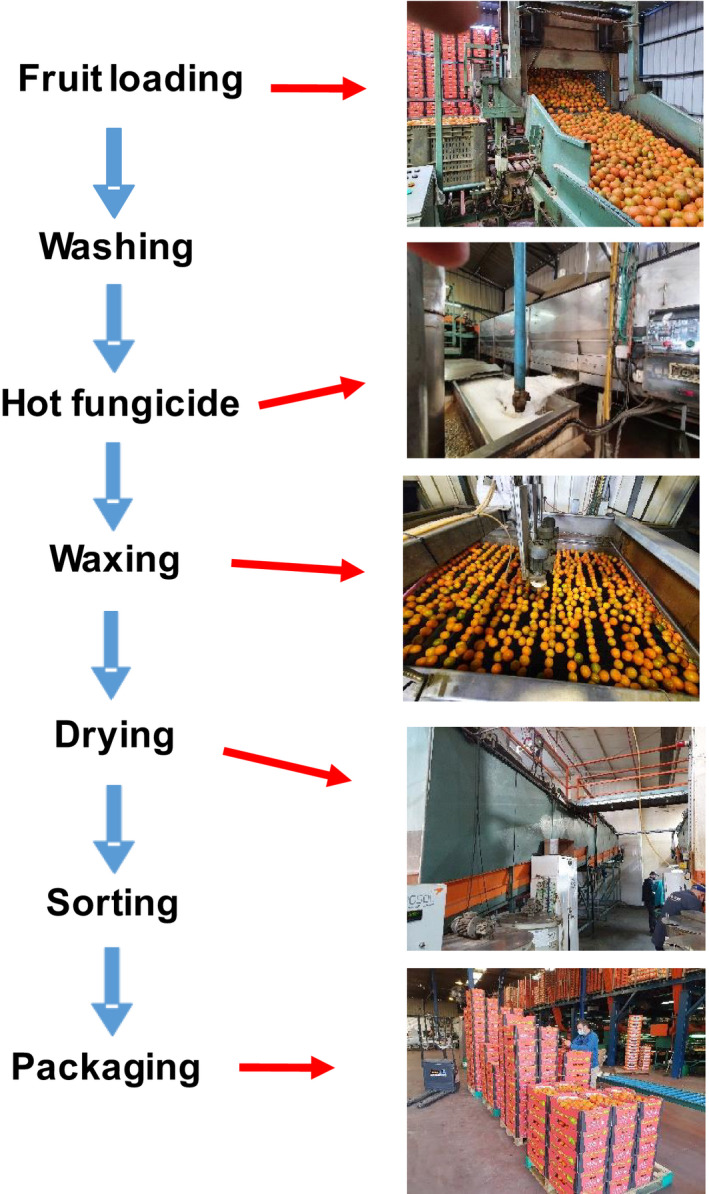
A schematic diagram of operations in a citrus packinghouse

After harvest, citrus fruit are transported to commercial packinghouses for further processing and packaging (Ritenour et al., 2006). Along the packing line, the fruit are subjected to various processes, including cleaning and washing, the application of fungicides and wax coatings, drying, sorting, and packaging (Figure 1). These processes are meant to improve fruit appearance and prevent postharvest deterioration. For example, washing with detergents and brushes cleans the fruit, the application of fungicides prevents decay, and waxing provides a shiny and attractive appearance and reduces water loss and shrinkage (Berk, 2016). Fungicides such as imazalil are often applied in a hot solution (~53°C), which increases their efficacy and further increases the fruit's ability to tolerate low storage temperatures (Ansari & Feridoon, 2007).

One of the most important interventions in commercial citrus packinghouses is the application of wax coatings, which impart shine, reduce water loss and shrinkage, and delay ripening and senescence (Petracek et al., 1999). Wax coatings are commercially applied by spraying the fruit as they move along a belt of brush rollers. Afterward, the fruit pass through a hot‐air drying tunnel (~37°C) for 2–5 min, to ensure the proper drying of the wax coating.

The overall flavor of citrus fruit is derived from the combination of taste, aroma, and mouthfeel sensations (Porat et al., 2016). More specifically, the taste of mandarins is mainly governed by the levels of sugars, acids, and bitter compounds. Mandarin aroma is mainly governed by the content and composition of aroma volatiles and mandarin mouthfeel sensation is mainly governed by the degree of juiciness and segment hardness (Goldenberg et al., 2015; Tietel, Plotto, et al., 2011).

Previous studies have attributed the development of off‐flavors in mandarins to the induction of ethanol‐fermentation metabolism and the accumulation of high levels of ethanol in the juice sacs (Cohen et al., 1990; Shi et al., 2007; Tietel et al., 2011). The accumulation of off‐flavors in mandarins is remarkably enhanced by the application of wax coatings, which restrict gas exchange through the peel surface, thereby stimulating anaerobic respiration and ethanol accumulation (Davis & Hofmann, 1973; Hagenmaier, 2002; Obenland & Arpaia, 2019; Porat et al., 2005; Tietel et al., 2010).

In addition to the direct effect of ethanol accumulation on perceived fruit flavor, it has been shown that, together with other acyl‐coenzyme As (CoAs), ethanol may also serve as a substrate for subsequent esterification reactions that lead to the accumulation of ethyl ester volatiles (Tietel, Lewinsohn, et al., 2011). For example, it has been reported that the levels of various ethyl esters, including ethyl acetate, ethyl propanoate, ethyl 2‐butanoate, and ethyl 2‐methyl butanoate, increase during postharvest storage of mandarins and the accumulation of such ethyl ester volatiles may also impact fruit‐flavor perception (Obenland et al., 2011; Ummarat et al., 2015).

The main goal of the current study was to examine the effects of commercial packinghouse operations on the flavor of ‘Orri’ mandarins. It is worth noting that ‘Orri’ mandarins bring in exceptional, high profits in export markets due to their excellent flavor quality and, therefore, the preservation of that flavor quality through processing and storage is of great commercial importance (Goldenberg et al., 2015).

## 2 MATERIALS AND METHODS

### 2.1 Plant material and packinghouse operations

‘Orri’ mandarins were collected from the Mehadrin Pri‐Or Ltd. packinghouse in Ashkelon, Israel. To examine the effects of packinghouse operations on fruit quality, we collected fruit at four different stages along the commercial packing line: (1) from the harvest bins, (2) after application of a hot fungicide treatment, (3) after waxing, and (4) after waxing and drying (Figure 1). The hot fungicide treatment included spraying the fruit for ~30 s with 400 µl L^−1^ of hot (53°C) imazalil and the drying treatment involved passing the mandarins through a hot‐air drying tunnel (kept at 37°C) for 2 min. Each treatment included three cartons of ‘Orri’ mandarins, with each carton containing 30 fruit. The fruit were coated with a commercial ‘Tag’ wax formulation particularly used for coating mandarins (DECCO SafePack, Hadera, Israel).

### 2.2 Postharvest storage

Within 1 h of the packinghouse treatments, the fruit were transferred to the Department of Postharvest Storage at the Volcani Institute, where they were stored for 3 or 6 weeks at 5°C, and then transferred for an additional 5 days of storage under shelf‐life conditions at 22°C. The relative humidity (RH) was ~95% during cold storage and ~80%–85% during the shelf‐life simulation. The fruit were stored in cartons without plastic wrappings.

### 2.3 Juice total soluble solids (TSS) and acidity

The total soluble solids (TSS) contents of the juice of the fruit exposed to the different treatments were determined with a PAL‐1 digital refractometer (Atago) and acidity percentages were measured by titration to pH 8.3 against 0.1 M NaOH with a CH‐9101 automatic titrator (Metrohm). Each measurement included four replications and each replication included juice collected from three different fruit (i.e., 12 fruit per treatment).

### 2.4 Juice ethanol levels

Ethanol concentrations in the juice were determined according to Davis and Chace (1969). In this experiment, 10‐ml juice aliquots were incubated at 37°C for 30 min in 25‐ml Erlenmeyer flasks. Similar Erlenmeyer flasks containing 10 ml of 100 µl L^−1^ ethanol were used as a reference standard for the calculation of ethanol concentrations. After the incubation period, 2‐ml gas samples were withdrawn from the flasks’ headspaces, using a syringe, and then injected into a Varian 3300 gas chromatograph (GC). The results presented are means ± standard error (SE) of four replicate samples; each replicate contained juice collected from three different fruit (i.e., a total of 12 fruit per treatment).

### 2.5 Analysis of aroma volatiles

Aroma volatiles of ‘Orri’ mandarin juices were extracted and analyzed, as described previously (Goldenberg et al., 2016; Tietel et al., 2010; Tietel, Lewinsohn, et al., 2011). One‐ml juice samples were placed in 10‐ml glass vials, together with an equal volume of 30% (w/v) NaCl solution and 0.6 g NaCl, to inhibit enzymatic degradation. The mixtures were then stored at −80°C until analysis. Each evaluation included five samples, each made up of the juice of three different fruit. Gas chromatography coupled with mass spectrometry (GC–MS) was used to identify aroma volatiles. Before the analysis, the frozen samples were thawed at room temperature and then allowed to equilibrate for 5 min at 40°C. Volatiles were extracted by solid‐phase microextraction (SPME) using a divinylbenzene/carboxen/polydimethylsiloxane (DVB/CAR/PDMS) stable flex fiber (Supelco). The extracted volatiles were injected using an auto‐sampler (CTC PAL) into the splitless inlet of a Model 7890A gas chromatograph (Agilent) equipped with an HP‐5 column (30 m × 0.25 mm i.d., 0.25 µm film thickness; J&W Scientific) by desorption for 2 min at 250°C. The oven was programmed to run at 50°C for 1 min, then to ramp up to 160°C at 5°C/min, then up to 260°C at 20°C/min, and, finally, to remain at that temperature for 4 min. The helium carrier gas flow was set at 0.8 ml/min. The effluent was transferred to a Model 5975C mass spectrometer detector (Agilent) that was set to scan the m/z range from 40 to 206 at 7.72 scans s^−1^ in positive‐ion mode and mass spectra in the electron‐impact mode were generated at 70 eV. Chromatograph peaks were identified by comparing the mass spectrum of each component with the US National Institute of Standards and Technology (NIST) 2006 Mass Spectral Library. Identification of aroma volatiles was further confirmed by calculating their linear retention indices using a series of n‐alkanes (C5–C20) and comparing their values with previously published values. The identification of 27 compounds was further confirmed by comparing their retention times with those of chemical standards (Sigma‐Aldrich). Volatile levels were calculated according to calibration curves and are represented as limonene equivalents.

### 2.6 Sensory evaluations

Sensory quality was assessed by conducting descriptive and acceptance tests according to standard procedures (Lawless & Heymann, 2010). In both cases, the fruit were peeled and fruit samples were provided to the tasters as cut‐separated segments placed on Petri dishes or plates. All samples contained cut segments from at least six different fruit and were assigned three‐digit identification codes. The fruit samples were prepared within 1 h before the sensory tests.

Descriptive sensory tests were performed by a trained sensory panel comprised of 10 members, five males and five females aged 25–62, who routinely performed flavor tests of citrus fruit (Goldenberg et al., 2015,2016). Each panelist evaluated various sensory attributes including ‘sweetness’, ‘sourness’, ‘bitterness’, ‘juiciness’, ‘difficulty to chew’, ‘fruity aroma’, and ‘off‐flavors’ on a scale of 1–9, in which 1 = ‘very low’ and 9 = ‘very high’.

Acceptance sensory tests were performed by a group of 30–35 tasters, who were employees or students working at the Department of Postharvest Science, ARO, The Volcani Institute. The flavor‐acceptance scores were evaluated according to a 9‐point hedonic scale, in which 1 = ‘extreme dislike’ and 9 = ‘extreme like’.

### 2.7 Statistical analysis

One‐way analysis of variance (ANOVA) and Tukey's honest significant difference (HSD) pairwise comparison tests were conducted using JMP statistical software version 15.0 (SAS Institute Inc.). Microsoft Office Excel was used to calculate means, standard deviations, and standard errors.

## 3 RESULTS

**FIGURE 2:**
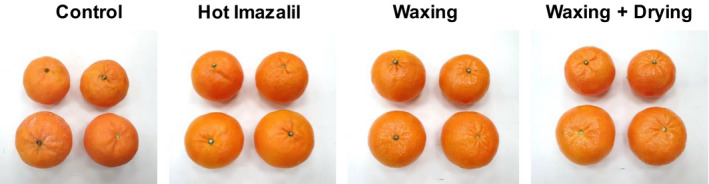
Photographs of ‘Orri’ mandarins collected from different points along the commercial packing line

Photographs of ‘Orri’ mandarins collected at the beginning of a commercial packing line and after the application of hot imazalil, waxing, and drying are presented in Figure 2. The untreated control fruit seemed dirty and pale and were less attractive than the hot imazalil‐treated fruit, which appeared to be nice and clean. In contrast, with or without the drying treatment, the waxed fruit seemed shinier and more attractive.

**FIGURE 3:**
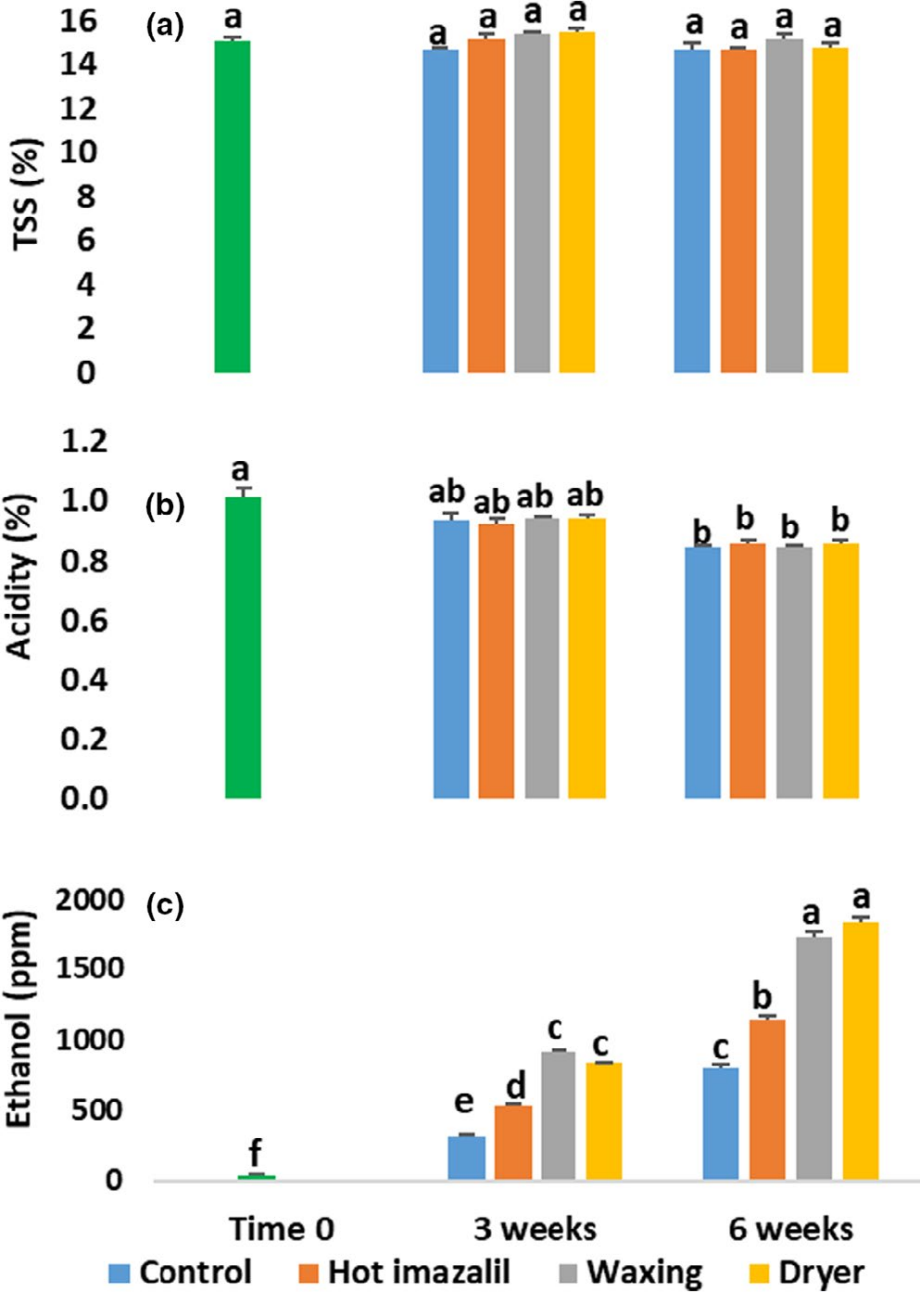
Effects of commercial packinghouse treatments on juice total soluble solids (TSS), acidity, and ethanol levels of ‘Orri’ mandarins. (a) TSS, (b) acidity, and (c) ethanol level. Measurements were conducted at Time 0 and after 3 and 6 weeks of storage at 5°C + 5 days at 22°C. Different letters indicate significant differences at *p* ≤ .05

Our biochemical analysis of the fruit juices did not reveal any significant differences in TSS levels, which remained stable between 14.7% and 15.2% in all treatments and for all of the evaluation periods (Figure 3a). Nonetheless, we detected gradual decreases in acidity from 1.01% at Time 0 to 0.92%–0.94% after 3 weeks of storage and 0.84%–0.86% after 6 weeks of storage (Figure 3b). Although there was a significant decrease in acidity after 6 weeks of storage, as compared to Time 0, we did not detect any significant differences among the different packinghouse treatments.

In contrast to juice TSS and acidity levels, we did detect dramatic increases in juice ethanol levels from just 36 ppm at Time 0 to 320–910 ppm after 3 weeks of storage and 800–1840 ppm after 6 weeks of storage (Figure 3c). The juice ethanol levels were significantly higher in all treatments after storage, as compared to the initial ethanol levels observed at Time 0. Nevertheless, for both storage periods, juice ethanol levels were significantly higher in the waxing and waxing +drying treatments than in the control and imazalil‐treated fruit.

**FIGURE 4:**
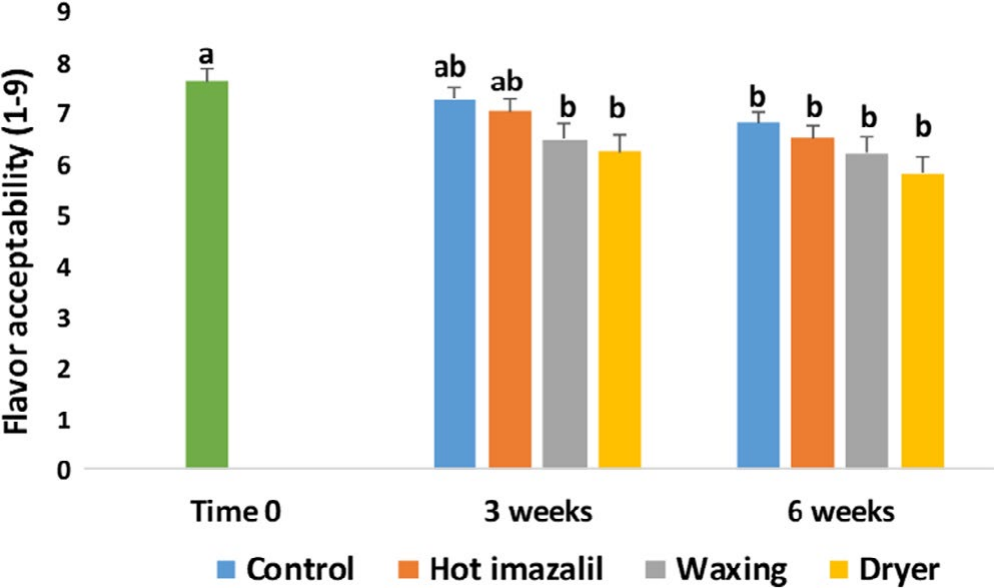
Effects of commercial packinghouse treatments on the flavor‐acceptability scores of ‘Orri’ mandarins. Sensory acceptance tests were conducted at Time 0 and after 3 and 6 weeks of storage at 5°C + 5 days at 22°C. Data are means ± standard error (SE) of the ratings assigned by 30 tasters. Different letters indicate significant differences at *p* ≤ .05

The flavor‐acceptance score of ‘Orri’ mandarins at Time 0 was 7.6 on a scale of 1 to 9, and it decreased to between 6.5 and 7.1 after 3 weeks of storage, and to between 6.0 and 6.7 after 6 weeks of storage (Figure 4). After 3 weeks of storage, we observed significant decreases in flavor acceptability only in the waxing and waxing +drying treatments; whereas after 6 weeks of storage, we observed significant decreases in all treatments. After 6 weeks of storage, the flavor‐acceptability scores of the waxed fruit were somewhat lower, but not significantly different from those of the control stored fruit.

**FIGURE 5:**
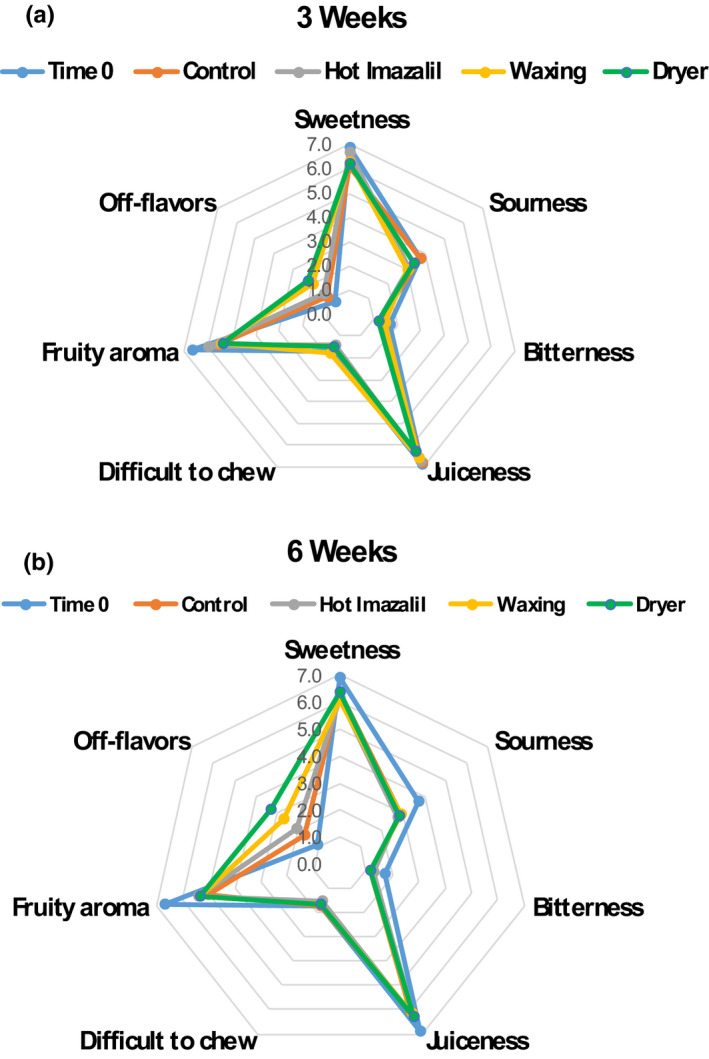
Effects of commercial packinghouse treatments on the flavor profiles of ‘Orri’ mandarins. Sensory descriptive tests were conducted with the aid of a trained panel at Time 0 and after (a) 3 and (b) 6 weeks of storage at 5°C + 5 days at 22°C. Data are means of scores assigned by 10 tasters

Descriptive sensory‐analysis tests conducted with the aid of a trained panel revealed that after the shorter, 3‐week storage period there were some decreases in the sensation of ‘fruity’ aroma in all treatments and slight increases in ‘off‐flavor’ sensation only in the waxing and waxing +drying treatments (Figure 5). After the longer 6‐week storage period, we observed decreases in ‘sourness’, ‘bitterness’, and ‘fruity aroma’, and parallel increases in the sensation of off‐flavors. After 6 weeks of cold storage, we observed only slight increases in off‐flavors in the control and imazalil‐treated fruit, as compared with much more pronounced off‐flavors in the waxing and waxing +drying treatments. Statistical analysis using analysis of variance (ANOVA) revealed that the increases in off‐flavor sensations in the waxing and waxing +drying treatments at both storage durations were significantly different (*p* ≤ .05) from off‐flavor sensation at time zero, and that off‐flavor sensation of the waxing +drying treatment was significantly different from the control fruit after 6 weeks of storage (data not shown).

**FIGURE 6:**
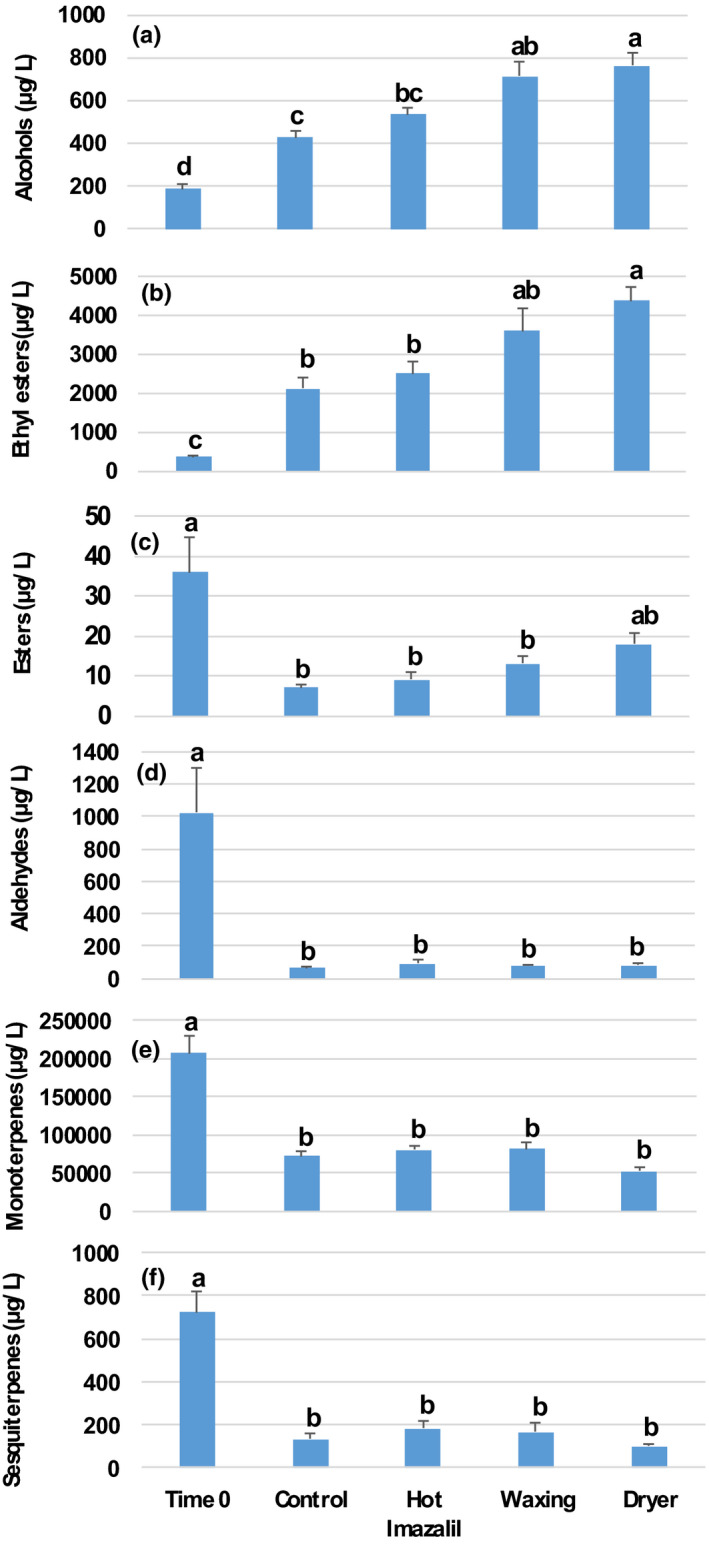
Effects of commercial packinghouse operations on the aroma‐volatile compositions of ‘Orri’ mandarins. The data represent the total levels of (a) alcohols, (b) ethyl esters, (c) esters, (d) aldehydes, (e) monoterpenes, and (f) sesquiterpenes. The analysis was conducted at Time 0 and after 6 weeks of storage at 5°C + 5 days at 22°C. Data are means ± SE (standard error) of five replications. Different letters indicate significant differences at *p* ≤ .05

We further examined the effects of the various packinghouse operations on the aroma‐volatile compositions of ‘Orri’ mandarins. Overall, through GC–MS analysis, we identified a total of 44 aroma volatiles, including 4 alcohols, 5 aldehydes, 10 ethyl esters, 2 esters, 3 terpene alcohols, 13 monoterpenes, and 7 sesquiterpenes (Table 1). We observed marked increases in the levels of alcohols and ethyl esters and marked decreases in the levels of esters, aldehydes, monoterpenes, and sesquiterpenes (Figure 6).

**TABLE 1:** Effects of commercial packing‐line operations on the aroma‐volatile compositions of ‘Orri’ mandarins

Compound			Concentration (µg L^−¹^)					Odor descriptor^e^
	RI^a^	RI^b^	Time 0	Control	Hot imazalil	Waxing	Drying	
Alcohols (4)								
Ethanol	‐	537	143	399	499	678	719	alcoholic
3‐Methyl butanol^d^	730	733	‐	‐	‐	2	4	roasted, wine, onion
2‐Methyl butanol^d^	733	739	‐	‐	7	9	11	fermented, fusel, fruity
Pentanol^d^	762	765	46	28	28	25	29	fusel, fermented, fruity
Aldehydes (5)								
Acetaldehyde^d^	‐	<500	13	26	26	25	26	pungent, solventy
Octanal^d^	1005	1002	588	32	37	38	25	aldehydic, waxy, citrus
Nonanal^d^	1107	1103	23	‐	13	‐	19	aldehydic, waxy, orange
Decanal^d^	1208	1203	361	10	17	18	13	aldehydic, waxy, citrus
Dodecanal	1412	1407	41	‐	‐	‐	‐	soapy, mandarin, floral
Ethyl esters (10)								
Ethyl acetate^d^	614	600	63	283	294	534	497	ethereal, fruity, sweet
Ethyl acetone	688	686	‐		2	3	10	ethereal, sweet, fermented
Ethyl propanoate^d^	711	714	‐	30	31	38	43	fruity, sweet, ethereal
Ethyl 2‐methylpropanoate	755	751	‐	9	10	14	18	pungent, ethereal, fruity
Ethyl butanoate^d^	802	794	210	1534	1844	2500	3273	fruity, fresh, ethereal
Ethyl 2‐butenoate^d^	842	823	‐	13	12	27	24	pungent, fermented
Ethyl 2‐methylbutanoate^d^	848	846	‐	25	25	40	41	fruity, fresh
Ethyl hexanoate^d^	1001	998	69	187	224	363	382	fruity, estery
Ethyl 3‐hydroxyhexanoate	1130	1126	1	10	10	13	12	sweet, fruity
Ethyl octanoate^d^	1199	1193	11	26	40	63	60	waxy, fruity
Esters (2)								
Methyl butanoate	721	724	‐	7	9	13	18	pungent, ethereal, fruity
Octyl acetate	1214	1208	36	‐	‐	‐	‐	floral, waxy
Terpene alcohols (3)								
Linalool^d^	1102	1100	180	27	29	28	21	floral, citrus, sweet
4‐Terpineol^d^	1182	1182	26	10	8	7	‐	spicy, woody, citrus
α‐Terpineol^d^	1195	1193	30	8	8	8	7	piney, terpene, citrus
Monoterpenes (13)								
α‐Thujene	929	930^c^	43	13	7	20	10	woody, green, herb
α‐Pinene^d^	936	936	3,127	606	662	819	377	herbal, woody, piney
Camphene	951	953	17	‐	6	4	3	woody, camphoraceous
Sabinene	976	976	791	131	167	167	82	woody, spicy, citrus
β‐Pinene^d^	980	980	87	111	22	22	12	herbal, fresh, piney
Myrcene^d^	994	991	11,303	2323	3288	3272	1687	spicy, herbaceous, citrus
α‐Phellandrene^d^	1008	1008	368	77	96	95	51	terpenic, citrus, green
α‐Terpinene	1022	1018	51	17	24	18	11	woody, citrus, terpenic
Limonene^d^	1044	1031	190,440	68,260	76,254	77,090	50,246	citrus, fresh, sweet
cis‐β‐Ocimene^d^	1052	1037	657	89	127	104	59	floral, herb, sweet
γ‐Terpinene	1063	1071	124	20	29	29	18	terpenic, sweet, citrus
Terpinolene^d^	1093	1091	174	46	52	53	27	herbal, sweet, citrus
Perillaldehyde^d^	1280	1279	40	‐	‐	‐	‐	aromatic, herbal
Sesquiterpenes (7)								
α‐Cubebene	1357	1,351	88	16	20	21	11	herbal, waxy
Copaene	1385	1,376	287	54	81	78	43	woody, spicy, honey
β‐Cubebene	1399	1,390	166	16	22	22	13	citrus, fruity, radish
β‐Caryophyllene^d^	1493	1,432	26		7			spicy, sweet, woody
Valencene^d^	1506	1,506	4	12	10	12	9	citrus, sweet, fresh
α‐Panasinsene	1511	1,530	27	13	7			
β‐Cadinene	1540	1,538	122	22	34	33	21	woody, green

Note

Analysis was conducted at Time 0 and after 6 weeks at 5°C + 5 days at 22°C. Data are means of five replications.

^a^ Calculated retention indices based on a series of n‐alkanes.

^b^ Published retention indices on DB‐5 column according to the University of Florida Citrus Flavor Database, unless mentioned otherwise.

^c^ Published retention indices on DB‐5 column according to Adams (2001).

^d^ Volatile identification confirmed with chemical standards.

^e^ Odor descriptions according to The Good Scents Company.

We observed significant increases in the alcohol levels in the control and imazalil treatments after storage, relative to the initial levels observed at Time 0, but significantly greater increases following the application of the waxing and waxing +drying treatments (Figure 6). It is worth noting that the majority of the observed increases in alcohol levels were due to increased ethanol content (Table 1).

Regarding the increases in the levels of ethyl esters, we observed significant increases in the control and hot imazalil‐treated fruit after storage, relative to the initial levels observed at Time 0, but significantly greater increases in the waxing and waxing +drying treatments (Figure 6). The majority of the observed increases in ethyl ester levels were due to increases in ethyl acetate and ethyl butanoate, which are formed through the esterification of ethanol and butanol, respectively (Table 1).

Regarding the ester levels, we observed significant decreases in all of the treatments, except for the waxing +drying treatment. More specifically, the levels of octyl acetate decreased after storage, while the levels of methyl butanoate increased after storage (Table 1).

In terms of the levels of aldehydes, monoterpenes, and sesquiterpenes, we observed significant decreases in all treatments after storage, without any significant differences between the control and the various packinghouse treatments.

## 4 DISCUSSION

Flavor is one of the most important fruit‐quality parameters and, therefore, it is necessary to optimize all fruit processing and postharvest operations to maintain flavor quality as best as possible (Kader, 2008). In this context, the main goal of the current study was to examine the effects of various commercial packinghouse operations, including the application of a hot fungicide, waxing, and drying, on the flavor of ‘Orri’ mandarins. The key finding of this research is that the packinghouse operation that most strongly affected mandarin flavor quality was the practice of applying a wax coating to the fruit, which imparted shine and reduced water loss, but also somewhat harmed fruit flavor and enhanced the development of off‐flavors (Figures 4 and 5). In this respect, the current findings are consistent with those of many previous studies, which have demonstrated that the application of wax coatings may harm mandarin flavor (Cohen et al., 1990; Davis & Hofmann, 1973; Hagenmaier, 2002). Worth notice is that the decrease in flavor acceptability of the waxed fruit as compared to control untreated fruit was difficult to detect by consumer acceptance tests involving untrained panelists. Nonetheless, the sensation of off‐flavor accumulation was more pronounced and detectable by conducting descriptive tests with the aid of trained sensory panelists (Figures 4, 5).

Analysis of the biochemical composition of ‘Orri’ mandarins following the various packinghouse treatments revealed that the examined treatments did not affect TSS or acidity levels, the sensations of sweet and sour tastes, or mouthfeel sensations, such as juiciness or difficulty to chew (Figures 3 and 5). However, the practice of wax coating strongly affected juice aroma‐volatile levels, as well as the sensation of off‐flavors (Figures 4, 5, 6). According to the observed results, the main biochemical effect of the waxing process was the stimulation of the accumulation of ethanol and ethyl esters during storage (Figures 3 and 6). Similar findings that packinghouse operations stimulate ethanol accumulation and enhance off‐flavor sensations, but do not affect TSS and acidity levels were previously reported for navel oranges (Obenland et al., 2008). In fact, Obenland et al. (2008) suggested that the deterioration of citrus fruit flavor after harvest is the result of a joint response to both storage duration and packing‐line operations and our findings are in agreement with that assumption.

The current findings suggest that waxing‐induced ethanol accumulation is the main cause for the deterioration of mandarin flavor after harvest. Therefore, in order to maintain flavor quality, we need to identify and develop new wax formulations that will be more permeable to gases and, therefore, less encouraging of anaerobic respiration and the buildup of ethanol and off‐flavors. In fact, we previously reported that dilution of the polyethylene solids and shellac concentrations in commercial citrus Tag wax formulations reduced ethanol accumulation and off‐flavor development in ‘Mor’ mandarins (Porat et al., 2005). It is worth implementing this suggestion, to better maintain mandarin flavor quality after harvest. Furthermore, it is still necessary to examine and evaluate other new wax‐coating formulations and, preferably, edible‐coating formulations, which are safer for humans and which may be more gas‐permeable than polyethylene‐ and shellac‐based waxes and, therefore, less likely to stimulate ethanol production and the development of off‐flavors (Miranda et al., 2021). Other possibilities for retaining mandarin flavor quality after harvest are simply to reduce the amount of wax applied to the fruit by either shortening the exposure time to the waxes or reducing the amount of wax sprayed above the conveyer belt, or even marketing fruit without any wax coating.

## CONFLICT OF INTEREST

The authors declare that they have no conflict of interest.

## ETHICAL APPROVAL

This study did not involve any human or animal testing.

## Data Availability

Data available on request from the authors.
